# Development of Wernicke’s encephalopathy long after subtotal stomach-preserving pancreatoduodenectomy: a case report

**DOI:** 10.1186/s40792-020-00982-y

**Published:** 2020-09-25

**Authors:** Chikanori Tsutsumi, Toshiya Abe, Tomohiko Shinkawa, Hideyuki Watanabe, Kazuyoshi Nishihara, Toru Nakano

**Affiliations:** 1 Department of Surgery, Kitakyushu Municipal Medical Center, 2-1-1 Bashaku, Kokurakita-Ku, Kitakyushu, 802-0077 Japan; 2 Department of Radiology, Kitakyushu Municipal Medical Center, Kitakyushu, Japan

**Keywords:** Wernicke’s encephalopathy, Subtotal stomach-preserving pancreatoduodenectomy, Thiamine

## Abstract

**Background:**

Wernicke’s encephalopathy (WE) is an acute neuropsychiatric disorder resulting from thiamine (vitamin B_1_) deficiency, frequently associated with chronic alcoholism and total parenteral nutrition without thiamine. However, only a few reports have focused on the relationship between WE and subtotal stomach-preserving pancreatoduodenectomy (SSPPD).

**Case presentation:**

A 71-year-old woman underwent SSPPD for an adenocarcinoma of the ampulla of Vater. Although there had been no evidence of recurrence, the patient was treated with antibiotics for cholangitis at 12 and 31 months, respectively, post-surgery. Thereafter, the patient presented with vomiting and disorientation 33 months after surgery. Although she was admitted and underwent closer inspection by a neurologist and a psychiatrist, the exact cause of these syndromes remained unknown. The psychiatrist measured thiamine concentration to examine the cause of disorientation. After 6 days, her level of consciousness worsened. Magnetic resonance imaging of the head showed symmetrically multiple abnormal hyperintense signals on fluid-attenuated inversion-recovery and diffusion weighted image, compatible with WE. An administration of intravenous thiamine was immediately initiated. After 8 days of the measurement of the thiamine level, the patient’s serum thiamine level was found to be 6 µg/mL (reference range, 24–66 µg/mL). Accordingly, the patient was diagnosed with WE. Shortly after starting the treatment, blood thiamine value reached above normal range with significant improvement of her confusional state. However, short-term memory and ataxia remained.

**Conclusions:**

Development of WE after SSPPD is uncommon. However, to prevent an after-effect, the possibility of development of WE after SSPPD should be recognized.

## Background

Wernicke’s encephalopathy (WE) is an acute neuropsychiatric disorder resulting from thiamine (vitamin B_1_) deficiency, leading to significant morbidity and mortality. It is characterized by a typical triad of mental status changes, nystagmus, and ataxia [1]. WE is frequently associated with chronic alcoholism and total parenteral nutrition without thiamine [2]. However, the relationship of WE with subtotal stomach-preserving pancreatoduodenectomy (SSPPD) has not been widely documented. We report an extremely rare case of WE developing 3 years after SSPPD.

## Case presentation

**Fig. 1:**
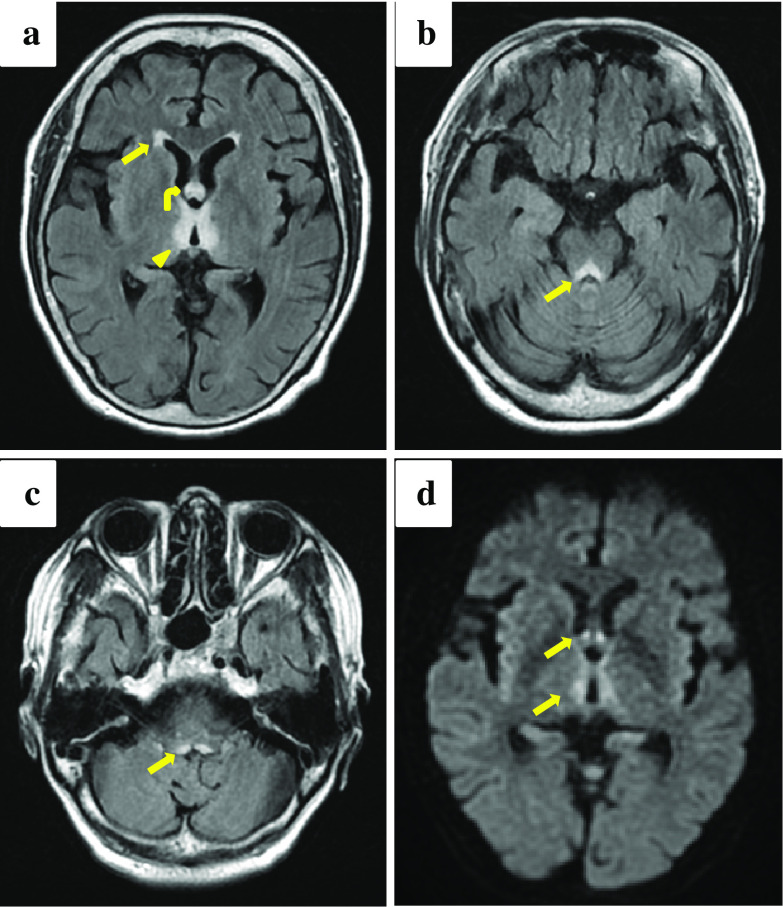
**a**–**d** Contrast-enhanced MRI of the head showing symmetrically abnormal hyperintense signal on FLAIR in the frontal horn of lateral ventricle (**a**, arrow), the medial thalami bordering the third ventricle (**a**, arrow head), the mammillary bodies (**a**, curved arrow), the periaqueductal region of the midbrain (**b**, arrow), and the bottom of the 4th ventricle (**c**, arrow). DWI imaging showing restricted diffusion corresponding to the FLAIR hyperintensities (**d**, arrows)

A 71-year-old woman underwent SSPPD for an adenocarcinoma of the ampulla of Vater, classified as stage IIB (pT1bN1M0) according to the 8th edition of the Union For International Cancer Control Tumor–Node–Metastasis classification. She experienced postoperative pancreatic fistula (PF) (Clavien–Dindo grade IIIa) [3], treated with drainage and antibiotics, and was discharged on day 21, postoperatively. At that time, laboratory data demonstrated that albumin and lymphocyte were 4.0 g/dL and 2050/µL, respectively, not suggesting malnutrition. After that, she had less oral intake than preoperative intake in addition to fasting for a month in total due to recurrent cholangitis. An adjuvant chemotherapy including gemcitabine and cisplatin were administered for 6 months. Post-chemotherapy, she was on regular follow-up every 6 months, without evidence of recurrence. She was treated twice with antibiotics for cholangitis at 12 and 31 months, respectively, post-surgery. She had lost 8 kg compared to preoperative body weight at the first cholangitis, whereas hypoalbuminemia (3.0 g/dL) and lymphopenia (820/µL) were present at the second cholangitis. She presented with vomiting 33 months after surgery. Additionally, her family complained of her disorientation. She was non-alcoholic, and had been able to eat until just before the onset of the symptom, except for the period of PF and cholangitis. On neurological examination, no specific findings were noted, including mental status despite her family complaint. She had not gained weight, since the onset of cholangitis. Laboratory data revealed hyponatremia (133 mEq/L), hypoalbuminemia (3.2 g/dL), and lymphopenia (810/µL), suggesting mild malnutrition. However, computed tomography (CT) and magnetic resonance imaging (MRI) of the head confirmed no specific findings. Although a neurologist and a psychiatrist examined closely, the exact cause of these syndromes remained unknown. The psychiatrist measured thiamine concentration to examine the cause of disorientation, whereas the laboratory measurement required 6–8 days. Thus, she underwent the treatment for hyponatremia until the results of thiamine value came. After 6 days, her level of consciousness worsened (Glasgow Coma Scale score, 3/15) [4]. Her comatose state made it difficult to evaluate other neurological findings. MRI of the head showed symmetrically abnormal hyperintense signal on fluid-attenuated inversion-recovery (FLAIR) and diffusion weighted image (DWI) in the frontal horn of lateral ventricle, the medial thalami bordering the third ventricle, the mammillary bodies, the periaqueductal region of the midbrain, and the bottom of the 4th ventricle (Fig. 1). As the MRI findings suggested WE, administration of thiamine (150 mg daily, intravenously) was immediately initiated. After 2 days, her serum thiamine level was 6 µg/mL (reference range, 24–66 µg/mL). Accordingly, she was diagnosed with WE. Shortly after initiation of the treatment, blood thiamine value reached 246 µg/mL, with significant improvement of her confusional state. After a week of intravenous therapy, oral administration of thiamine 100 mg daily was started. However, as short-term memory loss and ataxia remained, 3 weeks after starting the therapy, she was transferred to a rehabilitation hospital with continuation of thiamine administration. After 3 months, the memory impairment continued although walking with support was restored.

## Discussion

WE is an acute neurological syndrome resulting from a deficiency in thiamine (vitamin B_1_), presenting with gait ataxia, nystagmus, and mental-status changes [1]. The main cause of thiamine deficiency is chronic alcohol abuse. The other notable causes include chronic dietary lack (unbalanced diet or parenteral nutrition without thiamine), poor absorption or low intake (pyloric obstruction, celiac disease, recurrent vomiting, or gravidic hyperemesis), excessive intake of carbohydrates in relation to the supply of thiamine, or a greater need for nutrition (growth, exercise, pregnancy, or infection) [2]. Thiamine is a water-soluble vitamin absorbed in the duodenum and breaks through the blood–brain barrier [5]. Additionally, it is taken up in the body via the thiamine transporter and its expression is higher in the duodenum and stomach [6]. Thus, the duodenum and stomach are important for oral absorption of thiamine. Recently, preoperative nutrition status in patients after pancreatoduodenectomy (PD) was reported to be significantly correlated with the prognosis [7]. However, the correlation with WE and PD remains to be clarified. PD is a surgical procedure to resect duodenum and a part of stomach, reducing oral absorption of thiamine post-surgery. Although improvements in the operative technique and perioperative care have reduced mortality [8], the rate of postoperative complications of 40% or above is still high [9]. The complications such as PF, cholangitis, and bile leakage, can lead to poor oral intake (POI). Body’s store of thiamine is only sufficient for up to 18 days [1], and postoperative complications may further accelerate consumption of thiamine. The female in this case could not take anything orally for a certain period due to PF and recurrent cholangitis. In addition, laboratory data and loss of body weight suggested malnutrition at the time of onset. A search of the literature revealed only 11 patients developing WE after PD, including the present case [10–18] (Table 1). Almost all patients had aggravating factors such as POI, PF, and infections without tendency to develop WE in onset period. Shimomura et al. [19] stated that a late occurrence may be associated with a minor change in dietary habit precipitating a long-standing latent deficiency of thiamine. Therefore, POI due to PF and recurrent cholangitis might be related with late-onset WE in this case. In the present case, the development of WE may be explained by several causes, namely, the reduced duodenal and gastric mucosa useful for thiamine absorption, POI and increased requirement of thiamine secondary to PF and cholangitis, and minor change in dietary habit including decreased oral intake after SSPPD. Based on these findings, postoperative complications such POI, PF, and cholangitis after PD may be the risk factors for development of WE, in addition to thiamine malabsorption. Therefore, a combination of several factors may cause the development of WE after PD, although many cases who underwent PD did not develop WE.

**Table 1:** Preveous reports of WE patients underwent PD

No	Author	Year	Age	Sex	Main complain	Operation (diagnosis)	Time to WE	Aggravaiting factor
1	Tsujino [10]	2007	68	M	Pedel edema, foot numbness, ataxia	PD (AoV cancer)	8 years	Loop diuretic
2	Karayiannakis [11]	2011	52	M	Disorientation, ataxia	PD (pancreatic cancer)	1.2 years	Alcohol abuse, POI
3	Onieva-Gonzanlez [12]	2011	27	M	Disorientation, nystagmus, ataxia	PD (Duodenal ulcer bleeding)	Several days	Peumonia, septic shock, POI
4	Kilinc [13]	2015	38	F	Nystagmus, ataxia	PD (pancreatic cancer)	2 weeks	Obustraction, POI
5	AbdekRazek [14]	2018	54	M	Disorientation, nystagmus, ataxia	PD (pancreatic cancer)	5 years	POI
6	Ji Su Kim [15]	2018	65	M	Disorientation, nystagmus, ataxia	Laparoscopic PPPD (AoV cancer)	7 weeks	Bleeding, DGE, PF, POI
7	Sogabe [16]	2018	56	M	Pedal edema, disorientation, ataxia	PD (AoV cancer)	5 years	Not specified
8	Kanesada [17]	2018	72	F	Pedal edema, ataxia	SSPPD (pancreatic cancer)	4 years	POI
9	Monden [18]	2019	77	F	Disorientation, ataxia	PD (bile duct cancer)	4 years	PF
10	Present case	2020	71	F	Disorientation	SSPPD (AoV cancer)	3 years	PF, cholangitis, POI

*PD* pancreatoduodenectomy, *PPPD* pyorus-preserving pancreatoduodenectomy, *SSPPD* subtotal stomach-preserving pancreatoduodenectomy, *AoV* ampulla of vater, *POI* poor oral intake, *DGE* delayed gastric emptying, *PF* pancreatic fistula

The extent of gastrectomy is one-third in PD, whereas the stomach is largely preserved in SSPPD. Previous studies have reported most of the gastrointestinal surgeries such as gastrectomy and gastric bypass surgery as risk factors for WE [20, 21]. Furthermore, these studies demonstrated that there was no tendency in the timing of onset and most of surgical procedures were Roux-en-Y reconstruction or gastrojejunostomy. However, the reports focusing on the correlation with WE and SSPPD are scarce. In a literature search, there were only two cases of SSPPD, including the present case [17]. The development of WE after gastrointestinal surgeries and after PD had in common that food did not pass through the duodenum. Accordingly, WE may develop after SSPPD.

Early diagnosis of WE may prevent the transition to Korsakoff's syndrome (KS) [1]. However, the typical classic triad is present in only 20% of patients with WE [22] and a substantial proportion of WE patients are undiagnosed or misdiagnosed. The literature search [10–18] (Table 1) revealed that 30% of the cases had three typical symptoms. In this case, WE was not suspected despite her family complaining of disorientation. As MRI findings suggested WE, the disease was strongly suspected. Determination of thiamine concentrations in the blood is central to the diagnosis of WE. However, laboratory measurement of thiamine value requires 6–8 days. In addition, Weidauer et al. [23] demonstrated MRI with a sensitivity of 53% and a specificity of 93%, thus making the diagnosis of WE difficult. In the present case, second MRI revealed the suspicious findings of WE. This could be explained by the fact that WE got worse. On the other hand, lactic acidosis in WE results from failure of oxygen utilization due to mitochondrial dysfunction as thiamine is essential for mitochondrial metabolism [24]. Monden et al. [18] reported blood gas analysis including lactic acidosis to be useful for diagnosing WE. In the present case, we did not measure lactic acidosis. Moreover, thiamine deficiency can cause beriberi. Edema, a symptom associated with beriberi, was observed in 30% of the cases in the literature search [10–18] (Table 1). Therefore, in patients who underwent PD presenting with any of its typical symptoms or signs of heart failure, WE should be suspected and lactic acidosis may be useful for diagnosis.

WE, if left untreated, can progress to coma and even death. Furthermore, insufficient treatment leads to irreversible brain damage which may lead to death, with a mortality rate of about 20%, or to the chronic irreversible form of the encephalopathy in 85% of survivors [25]. However, there is no sufficient evidence to guide the clinician regarding the optimum dose, route, and duration of thiamine treatment. Although the therapy was initiated at the same time as WE was suspected, this patient was administrated thiamine 150 mg daily as large doses are not allowed under the Japanese insurance system. Unfortunately, short-term memory and ataxia remained. On the other hand, the European Federation of Neurological Societies guidelines recommend non-alcoholic WE patients to be treated intravenously with 200 mg thiamine thrice daily [26]. In this case, higher doses of thiamine may have prevented her after-effects. Further studies are needed to determine appropriate thiamine therapy for WE.

## Conclusions

Here, we report an extremely rare case of WE that developed 3 years after SSPPD. To prevent an after-effect, possible development of WE after SSPPD must be recognized.

## Data Availability

Not applicable.

## Abbreviations

WE: Wernicke’s encephalopathy
SSPPD: Subtotal stomach-preserving pancreatoduodenectomy
PF: Pancreatic fistula
CT: Computed tomography
MRI: Magnetic resonance imaging
FLAIR: Fluid-attenuated inversion-recovery
DWI: Diffusion weighted image
PD: Pancreatoduodenectomy
POI: Poor oral intake
KS: Korsakoff's syndrome
